# Medication Management Strategies to Support Medication Adherence: Interview Study With Older Adults

**DOI:** 10.2196/53513

**Published:** 2024-08-13

**Authors:** Lisa Gualtieri, Mathilda Rigby, Deelia Wang, Elaine Mann

**Affiliations:** 1 Tufts University School of Medicine Boston, MA United States; 2 Harvard Medical School Boston, MA United States

**Keywords:** home medication management, medication adherence, prescription drugs, adherence devices, adherence apps, pill cases, aging in place, independent living, aging, medication, older adults, prescription, interview, interview design, design, app, mobile phone

## Abstract

**Background:**

Home medication management has been insufficiently studied, including the factors that impact the development and effectiveness of adherence strategies under both routine and anomalous circumstances. Older adults are a particularly important population to study due to the greater likelihood of taking medication in combination with the desire to “age in place.”

**Objective:**

This interview study aims to understand how older adults develop medication management strategies, identify when and why such strategies succeed or fail, learn more about how older adults think about their medication, and explore interventions that increase medication adherence.

**Methods:**

This study used a qualitative, semistructured interview design to elicit older adults’ experiences with home medication management. Overall, 22 participants aged ≥50 years taking 1 to 3 prescription medications were recruited and interviewed. Interview responses were recorded, and thematic, qualitative analysis was performed by reviewing recordings and identifying recurring patterns and themes. Responses were systematically coded, which not only facilitated the identification of these themes but also allowed us to quantify the prevalence of behaviors and perceptions, providing a robust understanding of medication management and medication adherence.

**Results:**

Participants reported developing home medication management strategies on their own, with none of the participants receiving guidance from health care providers and 59% (13/22) of the participants using trial and error. The strategies developed by study participants were all unique and generally encompassed prescription medication and vitamins or supplements, with no demarcation between what was prescribed or recommended by a physician and what they selected independently. Participants thought about their medications by their chemical name (10/22, 45%), by the appearance of the pill (8/22, 36%), by the medication’s purpose (2/22, 9%), or by the medication’s generic name (2/22, 9%). Pill cases (17/22, 77%) were more popular than prescription bottles (5/22, 23%) for storage of daily medication. Most participants (19/22, 86%) stored their pill cases or prescription bottles in visible locations in the home, and those using pill cases varied in their refill routines. Participants used ≥2 routines or objects as triggers to take their medication. Nonadherence was associated with a disruption to their routine. Finally, only 14% (3/22) of the participants used a time-based reminder or alarm, and none of the participants used a medication adherence device or app.

**Conclusions:**

Participants in our study varied considerably in their home medication management strategies and developed unique routines to remember to take their medication as well as to refill their pill cases. To reduce trial and error in establishing a strategy, there are opportunities for physicians and pharmacists to provide adherence guidance to older adults. To minimize the impact of disruptions on adherence, there are opportunities to develop more durable strategies and to design aids to medication adherence that leverage established daily routines.

## Introduction

### Background

Medication adherence—defined as taking medication as agreed with one’s prescriber—is a crucial part of aging well. Studies have shown that ≥50% of US adults do not take their prescriptions as directed and that medication nonadherence is responsible for as many as 33% to 69% of hospital admissions and 125,000 deaths annually [1-3]. The World Health Organization emphasizes the importance of medication adherence, stating that “[i]ncreasing the effectiveness of adherence interventions may have a far greater impact on the health of the population than any improvement in specific medical treatment” [4].

There is a rising rate of medication nonadherence across all ages, sex, and race groups in the United States [5]. The greatest concern, however, is for older adults. According to population estimates by the United Nations World Population Prospects report, 1 in every 4 persons will be aged ≥65 years by 2050 in Europe and Northern America [6]. This represents a doubling in the older adult population—approximately 9% in 2019 to an estimated 16% in 2050. Aging populations require consistent medication adherence for health maintenance; approximately 67% of US adults aged 45 to 64 years take at least 1 prescription drug, and this figure rises to 88.5% for those aged ≥65 years [7].

Older adults are underrepresented in research on medication adherence and may face some unique barriers compared to the average population [8,9]. There is a consistently strong preference to “age in place” by older populations, preferring to remain within communities instead of institutional care [10]. However, home settings are associated with medication nonadherence [8,11]. Identifying barriers to medication adherence related to home medication management, specifically related to unintentional nonadherence, may lead to successful strategies to aid older adults who wish to age in place [12]. Forgetting to take medication is one of the most common contributors to unintentional medication nonadherence and is particularly relevant for older adults desiring to live independently [12].

Many interventions have been designed to aid in individual medication adherence, generally specific to a disease or treatment. Innovation in general prescription packaging, distribution, and education has been limited. Some innovations in container, labeling, and package design include blister packs, PillPack, and a variety of adherence devices ranging from digital pill containers to dispensing devices [13-16]. Studies have shown limitations to many of these innovations, particularly in their design and usability. These innovations have also had limited commercial success. Overall, no substantial impact on adherence has resulted from the use of simple adherence devices [17,18]. More than 700 medication adherence apps exist, but users often report technical difficulties such as schedule inflexibility because many do not take, or need to take, their medication at an exact time, as well as notification fatigue from daily timed reminders to take their medication [19,20]. Therefore, innovation in containers, packaging, devices, and apps represents potential targets for improvement of adherence [21-24].

Another way to improve medication adherence is counseling patients on home medication management practices [25]. Health care providers can play an efficacious role in encouraging behaviors to support adherence, especially ones tailored to patients’ medication regimens and needs [20,26]. External influences such as reduced time for medical appointments and the increased use of mail delivery of prescriptions, necessitating fewer pharmacy visits, reduce opportunities for medication adherence guidance from physicians or pharmacists, respectively.

Medication adherence often relies on the development of a behavior that is repeated in response to triggers and fits into a routine [27]. Triggers are actions that are taken or objects that are encountered that can help patients remember to take their medication. Habit formation is a main determinant of behavior change but can take trial and error to be established, especially when no guidance is provided by a health care provider or suggested from past caregiving experience [28]. Selection of triggers and formation of habits specific to medication management are understudied, especially regarding the factors that may impact the development and sustainability of strategies within the context of both routine and anomalous circumstances.

### Objectives

This study aimed to identify home medication management practices used by community-dwelling older adults with simple medication regimens. Specifically, this qualitative study aimed to (1) understand how older adults develop medication management strategies, (2) identify strategies that lead to adherence and when and why such strategies succeed or fail, (3) learn more about how older adults think about their medication, and (4) explore interventions that increase medication adherence.

## Methods

### Approach

On the basis of the results of a survey [29], we planned an interview study to learn in depth about the medication management experiences of older adults in their homes. Our goal was to better understand how older adults fit medication taking into their daily lives. Hence, our interest was in patients with simple regimens involving 1 to 3 medications because, with more, medication management is a more all-consuming daily task.

We developed an interview guide and semistructured interview process to elicit responses from older adults on their experiences with home medication management, including selection of medication storage location and development of medication-taking routines, to characterize which factors positively influence medication adherence when managing a relatively small number of medications. Many interview questions were based on survey results, which showed what respondents did but did not reveal why or how they made their decisions [29]. Therefore, the interview questions were designed to elicit a richer narrative of how and why home medication management decisions are made and patients’ perceptions about what worked and what did not. The protocol was piloted on Zoom (Zoom Video Communications, Qumu Corporation) with 1 individual in the study demographic to test timing, flow, and wording, and no changes were made. The interview guide is included in Multimedia Appendix 1.

The interview questions were developed to elicit information about how older adults manage their medications in their homes as part of the daily routine and under anomalous circumstances such as travel. Interview questions asked participants to recount their medication routines and were designed to cover the 3 components of adherence—initiation, implementation, and discontinuation—to fully address the stages of adherence [30].

For the initiation phase, participants were asked who prescribed their medications, whether this was during in-person or virtual visits, and if they visited a pharmacy or used mail delivery. Participants were questioned about the extent of their health care provider’s counseling or advice on home medication management, including how and where to store prescriptions and how to remember to take them. For the implementation phase, patients were asked about their daily home medication management practices, including how they administer, store, and identify their prescription medications. They were questioned about any vitamins or supplements they take. They were asked about any stigma they experienced about taking medication or about visitors to their home seeing their medication. In addition, participants were questioned about their strategies for ascertaining adherence, including alarms, apps, devices, and assistance from others, and their interest in the use of a medication adherence device that used sensors to alert them only when they forgot to take their medication. This device was described as using the same principle as a seat belt, chiming only when you both forgot to latch it and turn on the ignition in a car. The device was described as using one sensor on the participant’s prescription bottle or pill case and another sensor on a coffee pot, toothbrush, or another object related to the participant’s daily routine. Finally, in the discontinuation phase, participants were questioned about nonadherence. Demographic questions were asked at the end.

### Participants

Participants included older adults recruited in July 2022 through the Osher Lifelong Learning Institute (OLLI) at Tufts University [31]. Participants were recruited through postings in the weekly email newsletter sent to approximately 2000 OLLI members who subscribed. Inclusion criteria were participants aged ≥50 years, taking 1 to 3 prescription medications for chronic conditions (as opposed to taking on a short-term or an as-needed basis), proficient in English, and with no cognitive impairment.

A total of 22 people responded to the recruitment materials using a link in the OLLI newsletter. All the 22 respondents consented to the web form, responded to the email to schedule a time for an interview, and participated in the study. Ages ranged from 56 to 87 (mean 70.5, SD 6.3) years, and 82% (18/22) of the participants identified as female and 18% (4/22) as male. All participants were White, and all had a bachelor’s degree or higher educational status. All were community dwelling, living in a house or apartment alone or with a partner, and all lived in an urban or suburban setting in Greater Boston.

Despite the constraints imposed by a small sample size, data saturation was largely achieved. This was evident by recurring patterns and themes emerging in the responses and the ability to draw meaningful conclusions from our sample.

Semistructured qualitative interviews were conducted with 22 participants during August 2022. The study was conducted during the COVID-19 pandemic, and owing to physical distancing protocols, all interviews were conducted via Zoom teleconferencing software. The duration of each interview was 30 to 45 minutes. Consent to participate was obtained at recruitment, and consent to record the interview was obtained at the start of each interview.

### Interview Team

The interviews were conducted by 1 researcher (LG), who had many years of prior experience conducting interviews. Emails were sent to arrange an interview time, and at the start of each interview, participants were told that the purpose of the interview was for a research project on medication adherence and were asked to consent to recording the session. No bias, assumptions, or interests in the topic were reported about the interviewer. Another researcher (MR) attended all sessions but did not conduct any interviews. Both researchers (LG and MR) took informal notes during the sessions. Recorded sessions were transcribed verbatim.

### Ethical Considerations

Study protocols were approved by the Tufts University Health Sciences Institutional Review Board (STUDY00002865). All participants consented to participate in the interviews and were told the purpose of the research in the consent form. All participants were asked at the start of the interview if they would consent to recording the interviews, that they would be only identified by a participant number and their names or any identifying information would not be used, that all recordings would be securely stored and erased at the culmination of the research, and that they could skip any question they did not wish to answer. The recordings and all data were identified only by a participant number and were thus deidentified. All participants were compensated with a US $50 Amazon gift card, which was sent to their email address as a way of showing our appreciation for their time. This amount was deemed appropriate for 30 to 45 minutes without being coercive.

### Analysis

The transcript analysis aimed to explore and understand the experiences of the participants; therefore, thematic analysis was chosen as the analytic strategy. Thematic analysis is a qualitative descriptive approach that is used to identify, analyze, and report patterns within data and is useful for analyzing narratives [32]. Thematic analysis was performed by 2 researchers reviewing and coding recordings and transcripts. A subset of interview questions, deemed most relevant to uncovering home medication management practices, were coded as part of this analysis.

To initiate the analysis, the researchers reviewed the interview responses to identify prominent themes and noteworthy topics emerging from the data. After identifying potential areas of interest, a structured list of questions and topics was developed to serve as a preliminary coding framework for the analysis. The researchers independently reviewed the recordings and interview notes to apply the initial coding framework. They engaged in regular meetings to compare their findings and discuss any discrepancies. This collaborative approach ensured consistency and reliability in theme identification. It also allowed for the iterative refinement of the coding scheme through both deductive and inductive methods, as themes were allowed to evolve naturally from the data, while the initial framework provided a guide to maintain focus on the study’s aims.

Coding the responses facilitated the identification of significant themes and allowed the research team to quantify the prevalence of behaviors and perceptions, providing a robust understanding of medication management and medication adherence.

## Results

### Sample Description

Five themes were identified relevant to home medication management strategies. These themes corresponded to the stages of medication adherence: initiation, implementation, and discontinuation.

#### Theme 1: Participants’ Experiences of Obtaining Medications

The first set of interview questions asked participants how their medications were prescribed and obtained. The purpose of these questions was to start the interview with straightforward questions that were simple to answer yet relevant to this research. An additional objective was to ask participants if they received counseling or advice from a physician or pharmacist regarding home management of medication, including where to store them or how to remember to take them.

In response to being asked about who prescribed their medications, how they obtained them, and if any counseling or advice was provided, all the participants obtained prescriptions from their primary care physician or specialist, yet none received guidance from a health care provider about how to devise an effective medication management strategy. More than half of the participants (13/22, 59%) used trial and error to develop a strategy, which included trying different locations in the home or trying a pill case after seeing one in a local pharmacy. Five participants devised a strategy based on experience assisting someone else manage their medication. One participant who devised a strategy based on prior experience stated the following:

I have managed medications in the past for my mother and aunt, both of whom are deceased, but they had pill cases [which are] pretty common. It seems [like] it’s a good organizational tool. I didn’t think a whole lot about it [and] went out and got a pill case when I first started having prescriptions.

Participant 13

The remaining 4 participants devised their medication management strategy using suggestions from a friend or family member.

Approximately half of the participants (10/22, 45%) received prescriptions by mail delivery only, 14% (3/22) of the participants used a combination of mail and pharmacy pickup, and the remaining 41% (9/22) used pharmacy pickup only. All but 1 participant (21/22, 95%) received a 90-day supply of their prescription medication.

#### Theme 2: Participants’ Experiences of Taking Medications at Home

All participants (22/22, 100%) responded to the question, “Can you walk us through your daily schedule for taking your medications, specifically when you take your medications and where you store them?” All participants provided a description of their home management practices, which constituted unique routines. All participants reported on what they used for medication storage and where containers were placed, refill strategies for pill cases, and how they remembered to take their medication.

For medication storage, only 23% (5/22) of the participants kept their medication in the prescription bottles it was received in. Most of the participants (17/22, 77%) used pill cases to store their medication. Of these, there was considerable variance in the type of pill case used, including the number of compartments. Of the 17 participants, 7 (41%) used 1 weekly pill case with 7 compartments. For participants with morning and evening medication, 6% (1/17) used a 14-compartment pill case, while 29% (5/17) used 2 separate pill cases each week. Two participants 12% (2/17) used 2 pill cases each to be able to refill them together for a 2-week medication supply, 1 of them citing the inconvenience of refilling pill cases and the desire to do so as infrequently as possible. Referring to their pill case, one participant describes the following:

It’s kind of a pain to fill so I kind of put it off...but if it’s obviously empty [or if] there’s maybe one slot to go, I’ll say awesome, let’s just fill it...I’ve got some time.

Participant 5

One participant 6% (1/17) had a separate pill case for each of their medications, and another participant 6% (1/17) used a pill case that stored a 4-day supply of medication.

Most US pill cases are designed with “Sunday” on the left-hand side; hence, we expected participants to have a weekend refill routine; instead, there was variance in when pill cases were refilled and how participants remembered to refill them. Overall, 41% (7/17) of the participants relied on the visual cue of an empty pill case to refill it; however, this method did not typically lead to them refilling their case on the same day every week. One participant stated the following:

When I get to the point where something is empty, and I say, oh, time to refill. It should be every seven days, but sometimes I might forget. Or I don’t know why. But it doesn’t always work out to be every seven days. But anyway, whenever they’re empty, then they need to be refilled.

Participant 7

Furthermore, 59% (10/17) of the participants consistently refilled on a specific day of the week. One described the routine for refilling as follows:

Sunday morning, it’s a routine. After breakfast, I drag [my medication] out. I have two different sets [of pill cases]. So I always have one in reserve in the closet with the bottles of the pills. I can easily take [the medication] on Sunday. I don’t have to wait till I fill them in order to take pills [since] I already have a set ready. Usually after breakfast or when I have a chance during the day on Sunday, I’ll go ahead and fill the one that I’ve just emptied in the previous week and put it in the closet.

Participant 17

Participants using prescription bottles used 1 storage location for their currently used bottle; those who received a 90-day supply in multiple bottles used a secondary location for excess. Participants using pill cases used 2 storage locations: a primary storage location for the pill case itself and a secondary storage location for the prescription bottles used to refill the pill case. The primary storage locations used for prescription bottles and pill cases were visible locations in their home for 86% (19/22) of the participants. The kitchen table was the most common primary storage location (10/22, 45%), and the bathroom counter was the second most common primary storage location (4/22, 18%). For the 17 participants using pill cases, 14 (82%) used a secondary storage location that was hidden from sight. The most common secondary storage location was the kitchen cabinet (7/22, 32%), followed by the bathroom cabinet (5/22, 23%).

Independent of the storage container used, most of the study participants (20/22, 91%) took their medication during a time range tied to a routine, such as eating a morning meal, while only a small number (2/22, 9%) reported taking medication at an exact time every day. One participant reported taking her evening medication as follows:

Sometime after dinner and before going to sleep. It’s probably a two-, three-, or four-hour range there.

Participant 11

Only a few participants (3/22, 14%) used a digital time-based reminder or alarm to manage their medication. One participant set an Alexa device to give her an oral reminder to take her pill at 10 AM every day, 1 participant used a smartphone reminder, and 1 participant used an alarm. None of the participants used medication adherence devices or apps for reminders.

All participants (22/22, 100%) relied on at least 2 triggers to remind them to take their medication. No 2 medication-taking routines were identical among participants. Action triggers included eating a meal (10/22, 45%), getting ready for bed (5/22, 23%), and brushing teeth (4/22, 18%); object triggers included a visible pill case (17/22, 77%) and a water glass (4/22, 18%). Because most participants stored their medication in a visible area, they could see their medication container as they engaged in a routine; for example, of the 10 participants who took their medication during a meal, 8 (80%) stored their prescription bottle or pill case on a kitchen table or counter. Storing their medication in a visible area served as a second trigger, backing up the routine-based trigger. One participant recounted the following:

I have what I call my staging area, which is an area between my kitchen and my dining room. [My medication] stays in the [staging area] and since I take that medication right after dinner it’s right there. As I’m clearing the table, after I put the dishes in the sink, I just go and I take the medication right after dinner, and it’s visibly right there.

Participant 6

Some triggers were tactile, not just visible. One participant used the spatial orientation of her pill bottles to manage medication adherence, reporting the following:

I came up with a scheme, where I keep the medicines on one side of my microwave, or my toaster oven. When I take it, I put it on the other side.

Participant 4

While all participants relied on at least 2 triggers to remind them to take their medication, more than half (15/22, 68%) of the participants relied on ≥3 triggers. One participant who relied on 3 triggers, taking medication with a meal, using a pill case, and placing it on the dining table, missed the first trigger but saw their pill case, which acted as a fallback reminder.

#### Theme 3: Factors Contributing to Nonadherence

From the participants’ descriptions of their medication management, the use of ≥2 triggers served as “a safety net” most of the time, providing multiple reminders to take medication. Yet even multiple triggers failed at times. The most reported reason for medication nonadherence among participants was an unplanned or unexpected change of routine (13/22, 59%), such as missing breakfast, waking up later in the day, or being distracted by a phone call. Such events typically led to the absence of, or overlooking, a specific trigger. For example, a participant who relied on eating breakfast as a cue reported forgetting to take her medication if she skipped breakfast:

If I have to go somewhere, first thing in the morning, that’s a typical time when I forget. Because sometimes I don’t even have time for breakfast or for one reason or another didn’t get around to it. Then the next day, it’s Monday, but I’m looking at the Sunday [compartment of the pill] case saying, “Oh, I guess I forgot to take it yesterday.”

Participant 7

Another participant acknowledged difficulties when her usual routine was interrupted or altered, reporting the following:

I just got distracted. I was on the phone with a friend. [Forgetting medication is] more apt to happen if I’m with my mom or somewhere other than in my own home because I’m out of the routine, even though I have the pill with me.

Participant 17

The second most reported reason for nonadherence was travel. The 8 participants who reported nonadherence during travel explained it by citing a change in schedule, not being able to store their medication in the same place, or not having the same triggers in their usual routine available. One participant recalled the following:

I have occasionally forgotten. Frankly, it’s when I’m on vacation; even though I have them in the [weekly pill case], my routine is different on vacation. It [the pill case is] not in my kitchen on vacation. I’m away someplace.

Participant 19

Another participant reported forgetting to pack his medication:

There was one time, I remember, when I left my pills home. So there were like three days where I was not taking the pills.

Participant 18

Stigma was not reported as a contributor to nonadherence. No participants reported experiencing stigma about taking medication or about visitors to their home seeing medication. When asked about stigma, respondents spoke about how common it was for older adults they knew to be on medication, which, to them, eliminated the experience of stigma or embarrassment.

#### Theme 4: Perception of Medication

Participants were asked about how they thought about their medication, for example, prescription name, pill purpose, or pill appearance. In response to this question, approximately half of the participants (10/22, 45%) thought about their medication by the chemical name, with the next most popular response being by the appearance of the pill (8/22, 36%). A smaller number (2/22, 9%) thought about the medication’s purpose or the medication’s generic name. One participant described medications as follows:

I can’t name the one that I have been taking the longest, which is upstairs in the bathroom. [The medication for] underactive thyroid I just remember by the name Levoxyl. And I’ve always remembered it. The one downstairs I just recently started and it’s a statin. I still don’t know what the name of it is. But it’s just for cholesterol.

Participant 14

Another participant described medications as follows:

I know all of them by their generic names. And when they’re new, I think about what they’re meant to do. But over time, I recognize them by their shape and color. On a day to day basis, I probably look for the shapes. And it really is off-putting when the pharmacy either changes the generic or I change my insurance plan and deal with the pharmacy benefits manager who happens to have a different generic.

Participant 20

Furthermore, of the 19 participants who took vitamins or supplements in addition to their prescription medication, 18 (95%) treated their medications the same, not distinguishing between what was prescribed by a physician, recommended by a physician, or something they were taking independent of their physician. These 18 participants stored their vitamins and supplements in the same pill case as prescription medication, and they integrated them into their medication routine, rather than distinguishing between their prescription medications and their vitamins and supplements.

#### Theme 5: Interest in Adherence Device

The final question to participants was about their interest in the use of a medication adherence device that used sensors to alert them only when they forgot to take their medication. Of the 22 participants, 17 (77%) were interested in using this device; 12 (71%) participants expressed interest in using the device immediately, whereas 5 (29%) were open to using the device if their medication routine got more complicated or they experienced any cognitive decline. Of those 5 participants, 1 (20%) talked about the need to use a device in the future, saying the following:

At this point now, I wouldn’t [be interested in a device], because I just don’t need it. But certainly, if I was struggling to remember to take them or if I had, like some people I know, this very, very complex regimen. So, I would be open to it at some point, but not now.

Participant 9

Out of the 5 participants who were not interested in the device, 3 (60%) explained that they saw no need to change or add to their current routine, while 2 (40%) rejected the idea due to the potential of notification fatigue or an unwillingness to use technology for medication adherence.

## Discussion

### Principal Findings

The purpose of this qualitative study was to explore the experiences of older adults managing their medication in their homes. In conducting this study, our most significant finding was the complexity and uniqueness of what participants did to manage simple medication regimens. Another significant finding was the extent to which trial and error or prior experience were used to develop strategies without guidance from health care professionals.

Medication management strategies need to encompass how people think about their medication and not the artificial demarcation of prescription medication only. Study participants who took vitamins and supplements thought about their medication as prescriptions, vitamins or supplements recommended by physicians, and vitamins or supplements recommended by a friend or another source.

Another finding was the variability in the use of pill cases, including the number and type of pill cases used and the frequency and timing to refill them. Related to this was the complexity of medication storage location selection for both primary storage, for example, a storage location accessed daily for medication, and secondary storage, for example, storage used for extra medication supply. Overall, the sheer variability in home medication management strategies was surprising and unexpected, especially for participants with relatively simple medication regimens.

Additional results from each theme are discussed in the subsequent sections.

### Theme 1: Participants’ Experiences of Obtaining Medications

All study participants expressed that they received no guidance from a physician or pharmacist on any aspect of home medication management, including how to establish a routine to be adherent. Of the 22 participants, 13 (59%) designed their own medication management regimens. A small number relied on prior experience helping someone else manage their medication or through the advice of a friend or family member. This lack of guidance from health care providers presents a missed opportunity to increase medication adherence from a trusted professional [30]. A survey conducted by Gualtieri et al [29] found that 96% of middle-aged and older adult respondents were receptive to receiving guidance from a physician or pharmacist regarding their medication management. This guidance could occur as part of prescribing or a medication review by a physician or by a pharmacist during prescription pickup.

With more than half of participants receiving prescriptions by mail only (10/22, 45%) or a combination of mail and pharmacy pickup (3/22, 14%) and approximately all receiving a 90-day supply of their prescription medication, fewer pharmacy visits are required as part of obtaining prescriptions. While this may decrease the likelihood of running out of medication, it also serves to decrease the need to enter a pharmacy location and therefore may reduce the extent to which an older adult establishes a relationship with or asks questions of a pharmacist. Less time in a pharmacy may limit opportunities to explore adherence-related tools, such as pill cases, which are often displayed next to or near pharmacies in retail stores. Less explored consequences of the rise in mail delivery may be theft or degradation of medicine by extreme temperatures, rain, or humidity.

### Theme 2: Participants’ Experiences of Taking Medications at Home

Participants described a wide range of routines for taking their medication. Each routine was unique with varying degrees of complexity. Most integrated multiple tactile and visual triggers into their daily routines to prompt them to take their medication each day. These adherence regimens may be so varied due to a lack of physician or pharmacist guidance or established norms for home medication management. Additional diversity was found in the unique locations where participants stored their medication and the timing with which they refilled it.

When participants described their experiences of home medication management, they included how they stored their pills. Pill cases were the most popular medication adherence device used at home. A possible reason for the high prevalence of weekly pill cases is that they provide direct feedback on whether someone took their medication using visual cues. Whether the slot in the pill case is empty or full is a straightforward indicator, unlike a prescription bottle, where users have no way of knowing if they took a pill that day unless they count pills. Weekly pill cases are also the most commonly seen medication adherence tool at pharmacies, though as evidenced by the interviews, the style and how they are used can vary greatly. This theory aligns with other literature, which demonstrates that pill boxes can effectively aid medication adherence. Another study on medication adherence found that some patients favored using pill boxes for managing their medications because they provided a visual reminder that they had taken their doses [33].

The most common primary storage location for medication was the kitchen, which was associated with using food preparation or consumption as a trigger for medication taking. This may be because mealtimes are a stable component of a daily routine. Another possible factor explaining the popularity of a kitchen storage location is that the participants enter the kitchen to complete specific tasks, as opposed to using it for long periods. This prescription bottle or pill case may act as a visual cue in the kitchen.

### Theme 3: Factors Contributing to Nonadherence

Participants, in describing their medication management strategies, referred to the use of multiple triggers that served as reminders to take their medication. Because triggers sometimes fail, they may depend on their specific context. An object or action without its usual context might be less effective as a reminder.

When asked to recount the last time they were nonadherent, participants described a disruption leading to a change in routine in their home or being in a different location due to travel. Because the recounted occurrences are events that are not under the control of the participant, developing a robust medication management strategy should ideally accommodate these anomalies. During a change in routine, a trigger that is usually relied upon may be absent. More robust routines with multiple triggers may endure disruptions better, or more durable triggers that are not disrupted by unexpected events are needed. More planning may also be needed to accommodate disruptions and unplanned events. Adherence devices may provide reminders to reduce nonadherence under anomalous circumstances.

Our study pinpointed factors contributing to nonadherence that are consistent with those identified in an interview study by Mickelson and Holden [34]. Their findings highlighted that disruptions in daily routines or travel could result in lapses in medication adherence. In addition, they observed a variety of tools and technologies being used to manage medications, including pill boxes. Uniquely, their study also revealed that stigma played a role in nonadherence; participants were hesitant to be seen as sick, leading them to skip medications in social settings, a finding that contrasts with our study [34]. Our participants noted that medication use among older adults is so common that it does not carry any stigma for them.

### Theme 4: Perception of Medication

How participants referred to their medication varied considerably, with most referring to their medication using the chemical name, followed by appearance. The use of chemical names could be due to education because all participants had obtained a bachelor’s degree or higher. Another factor influencing how people refer to their medication could be the number of medications that they take; it may be harder to keep track of chemical names as the number of medications increases, especially with the complexity of chemical names.

The second most common mode of reference participants used was appearance. When pills are similar in appearance, concerns about medical error arise. It is worth investigating the correlation between how people think of their medication and medication adherence and if discussion with a physician or pharmacist should emphasize the medication name, appearance, and purpose to aid in accurate identification by patients, especially for tasks such as filling a pill case. The primary concern for medical error may be when a patient who relies on appearance for prescription identification receives a new generic or a dosing change and the new pill is a different shape, size, or color.

Most participants thought of their medication as prescription medication, vitamins, and supplements, without categorizing differently what they were prescribed, recommended by a physician, or took of their own volition. Adherence is more critical for prescription medications, but strategies to guide patients should ideally address how they think about their medication regimen and, for pill case users, how they select, use, and refill pill cases.

### Theme 5: Interest in Adherence Device

Only 1 participant currently relied on a general, as opposed to adherence, technology to assist with medication; this participant relied on a daily reminder from Alexa. None of the study participants currently used adherence devices or apps to generate reminders. More than two-thirds of the participants (17/22, 77%) expressed interest in using a device to assist with adherence only when needed, in contrast to devices with timed reminders. Only 2 individuals expressed a lack of interest in this technology. In addition, most study participants lived alone without others to remind them to take their medication, providing an opportunity where an adherence device might be beneficial as a backup. A lack of experience, positive or negative, with medication adherence device or app use did not seemingly deter participants from expressing interest in a device that integrated naturally into their routine.

### Strengths and Limitations

The strength of participant recruitment through the OLLI at Tufts University was that our recruitment goal of ≥20 participants was met quickly. However, the disadvantage was that our sample was not representative of the US Census for adults aged >50 years. The narrowness of our study population, due to our recruitment strategy, is a limitation of this study. A subsequent interview study was conducted with adults who identify as racial or ethnic minority individuals.

While our cohort consisted of participants who were more educated, and hence likely to have higher health literacy skills, they still experienced unintentional nonadherence. A higher level of education is associated with higher socioeconomic status; however, none of the strategies deployed for medication adherence by participants were costly. Furthermore, many participants were not working full time or were retired, which may influence their daily routines and their ability to travel. Because most of the participants lived alone, they were less likely to be able to rely on others to remind them.

Finally, participants were limited to those taking 1 to 3 medications and experiencing no cognitive decline. A more representative sample of older adults should include those taking more medications, including those with complicated schedules. It should also include those who are experiencing minor cognitive decline yet are living independently because this might add additional barriers to medication adherence strategy development and may increase forgetfulness.

Despite some limitations in the diversity of participants, this study has strengths in uncovering home medication strategy development and execution through the set of questions eliciting participants’ experiences. Face-to-face interviews lasting 30 to 45 minutes through Zoom enabled us to gain more insight and details than we would have discovered through a survey; in fact, our prior survey work led to the design of an interview guide to gain a deeper understanding. Participants shared freely how they developed and executed a home medication management strategy, when the strategy was successful, and when and why it failed.

### Future Directions

Future work will build on the results of this study as well as the completed interview studies on racial and ethnic minorities. One goal is to create educational interventions that lead to improved adherence under routine and anomalous circumstances and eliminate or reduce the trial and error process so often used. Ideally, an educational intervention would be delivered by physicians when prescribing, by pharmacists when dispensing, or by either professional when nonadherence is indicated by a patient or by refill frequency.

Related to this, and in support of patient-physician communication about adherence, another goal is to develop a scale for home medication management, much like those for medication adherence but focused on home practices. This scale could be used by physicians when prescribing medication to determine how likely a patient is to be adherent based on their current lifestyle or by pharmacists or family caregivers to provide guidance in developing an individualized medication management strategy.

Another goal is to design and test medication adherence devices that take a failsafe approach to helping older adults live independently longer. While current medication adherence devices and apps rely on time-based triggers, our study shows the reliance on routine, leading to our interest in routine-based triggers. Our study found that participants managed their medication in the context of a routine, yet their routines did not take place at an exact time but rather upon rising or in the morning. Alerts or reminders set for a specific time may be disruptive if the timing of the routine is variable. They can be even more confusing if the person has already taken their medication but still receives a reminder to do so. This may result in notification fatigue or in turning off notifications altogether. Thus, we plan to design and test a medication adherence device that reminds users to take their medication only when their current routine fails them and is robust enough to accommodate disruptions. Given the high interest from our participants when asked about this new medication adherence device, this has the potential to reduce unintentional nonadherence in older adults who are aging in place.

Given that study participants’ medication management strategies treated prescribed medication the same way as vitamins and supplements, we will further explore the implications of this for adherence. Home medication management strategies, to be effective, should align with how patients think about their medication. This should include any medication, prescription, or nonprescription that patients take. Related to how people think about medication is further pursuit of the implications of identifying medication by name, appearance, or purpose. The special concern is if there are transitions due to a change to a generic or in dosing and the ensuing challenges when refilling pill cases.

Related to this, a future study will seek to better understand the selection and use of pill cases over prescription bottles and the impact on remembering to take medication as prescribed, not taking extra doses when unsure if a dose was taken, and making refilling a case easier and more convenient. For both users of pill cases and prescription bottles, we aim to research how to support the development of more durable, disruption-proof triggers that function under routine and anomalous circumstances.

A final interest stemming from this study is the influence of home environments. We noted that participants who lived alone could develop a medication routine with substantial triggers with no limitations in terms of where they could place medication. An open question is whether this affords more flexibility to optimize the placement of a pill case or prescription bottle. Yet these same individuals lack someone in the home to remind them to take their medication, which for some provides an additional safety net. Another question is the influence of home size and the number of levels in a home in developing a strategy, in particular, how much proximity is needed between storage locations and heavily used locations in the home.

### Conclusions

The findings of this study provide important insights into the challenges older adults face in managing their medication and indicate opportunities to improve medication adherence in older adults. Our study participants formulated home medication management strategies without physician or pharmacist guidance, which represents an unexplored opportunity for improving adherence strategies through patient education provided by health care professionals during discussions with patients. Another opportunity is to reduce the extent to which trial and error is used to develop and refine medication management strategies through tailored guidance. Individualized guidance should include identifying more durable, disruption-proof triggers that function under routine and anomalous circumstances, including sufficient portability to accommodate travel. This qualitative study suggests that, in addition to supporting older adults who occasionally forget to take their medication, there are opportunities to improve adherence guidance to older adults when they are receiving their first long-term prescription or during a regimen change. It also suggests pathways for designing better adherence aids that integrate with established daily routines.

## Appendix

Multimedia Appendix 1 Interview guide.

## Abbreviations

OLLI: Osher Lifelong Learning Institute

## Data Availability

The data sets generated and analyzed during this study are available from the corresponding author on reasonable request.
