# Supplementary material for: Medication Management Strategies to Support Medication Adherence: Interview Study With Older Adults
Source: Interact J Med Res. 2024 Aug 13;13:e53513. doi: 10.2196/53513 (PMC11350294; doi:10.2196/53513)
Supplement: Multimedia Appendix 1 [file ijmr_v13i1e53513_app1.docx]

**Appendix I: Interview Guide**

1. Introduction Script: We are conducting a study on how people store their prescriptions and how they remember to take them. We are not interested in specific medications or conditions but are hoping to gain a better understanding of your medication habits, storage, and routine. You can skip any questions that you do not feel comfortable answering. As a reminder, we will be recording this session for our own purposes. In order to maintain your privacy, no identifying information will ever be disclosed, and all records will be on secure servers and will be deleted upon the completion of this study.
2. Obtaining Medications (Initiation):
   1. Physician
      1. Who prescribes your prescription medications? Does a physician or specialist prescribe your medications?
         1. Prompt: do you go to a doctor or healthcare provider? Do you order online?
         2. Rationale: we can find out how they are getting their prescriptions
      2. Are your medications prescribed during online or in-person doctor’s visits?
         1. Prompt: do you go into a physical office to see your doctor, or are you connecting on the computer or phone?
         2. Rationale: are people more or less likely to have discussions with their doctors in-person or online?
      3. Have you ever been offered counseling or advice by your physician about where in your home to put your prescription medications or how to remember to take them?
         1. Prompt: did somebody advise you about where to store your medications or how to increase your medication adherence?
         2. Rationale: There is nothing in the literature showing that providers give advice, but perhaps some do mention storage or adherence.
      4. Has your physician prescribed or recommended vitamins or supplements?
         1. Prompt: Has your physician said you need to take Vitamin D or another over-the-counter vitamin or supplement?
         2. Rationale: Patients may not think of this differently than prescriptions from an adherence perspective.
      5. Has the COVID-19 pandemic affected your experience with your physician?
         1. Prompt: Do you talk to them more/less regularly, feel more/less comfortable scheduling an appointment? Do you feel like your conversations or frequency of interactions with your physician changed during COVID?
         2. Rationale: The role of a physician also can play a part in adherence by both prescribing pills and facilitating conversation about a patient’s medication and their adherence.
   2. Pharmacy
      1. Where do you pick up your prescription medications? At the pharmacy or somewhere else?
         1. Prompt: Curbside pick-up? Drive through? At the pharmacy?
         2. Rationale: a drive through or curbside pick-up is less likely to facilitate a conversation between the pharmacist and patient about how to best take their medication or to answer any of the patient’s questions
      2. When you go to the pharmacy to pick up your medications, do you have a discussion with the pharmacist regarding the medication you are picking up? If yes, do you regularly see a specific pharmacist, and if yes, do you have discussions about your medication history/status?
         1. Prompt: do they tell you where to store it or what time you should take it? Do they give you any warning about side effects?
         2. Rationale: follow up question to the previous question
      3. Has the COVID-19 pandemic affected your experience with pharmacies and/or pharmacists?
         1. Prompt: did you switch from in-person pick-up to drive-through? Do you go to the pharmacy less?
         2. Rationale: important to consider how COVID affects how patients' normal routine of getting their medication
   3. Miscellaneous
      1. Has there been any point where you wish you would have had counseling or advice from a physician or pharmacist regarding your medications and how to take them or where to put them?
         1. Prompt: Have you felt unsure about when to take your medication or if you are storing it in the optimal location?
         2. Rationale: Understand receptivity to advice
3. Taking Medications at Home (Implementation):
   1. Type of Medication
      1. Can you walk us through your daily schedule for taking your medications, specifically when you take your medications and where you store them? Are there any specific conditions for taking your medications (i.e., with water or on a full stomach, etc.)
         1. Prompt: do you store them on your nightstand, fridge, kitchen table….? Do you take it with food at breakfast? lunch? dinner?
         2. Rationale: Understanding routines may help us develop a device using context-sensitive cues
      2. When obtaining medications, do you typically obtain a 30-day or a 90-day prescription?
         1. Prompt: Does your insurance, pharmacy, or doctor decide how many pills you receive?
         2. Rationale: 30-days versus 90-days would impact the size of a user’s storage of medication and how frequently a patient needs to refill medication
      3. What do you call your medications, such as by the prescription name, by the purpose, or by the appearance?
         1. Prompt: Do you think of it as the green pill, my heart pill, or by the actual name?
         2. Rationale: Does this correlate with health literacy or adherence in any way? What does it mean for device design that uses voice?
      4. In addition to these medications you mentioned, do you have any vitamins, supplements, or over-the-counter medications that you take on a regular basis?
         1. Prompt: Do you take vitamins or other supplements? examples could include iron or vitamin C.
         2. Rationale: We want to learn if people distinguish between their prescription medications and vitamins/supplements.
      5. Do you store your medications based on any climate conditions at your home such as humidity, temperature, or light?
         1. Prompt: do you have locations you use like a refrigerator, or ones you avoid like a steamy bathroom?
         2. Rationale: people may store their medications based on factors besides convenience.
      6. Do you own any pets or have any children? If so, have they ever impacted how or where you store your medications?
         1. Prompt: do you make sure your medication is out of reach and/or sight from your children/pets by storing it in a medicine cabinet, locked area, or high shelf?
         2. Rationale: children or pets may affect adherence to medication by not allowing the patient to store it in the optimal location
   2. Selecting a Location + Establishing a Routine
      1. Earlier you walked us through your medication regimen. Please tell us all the locations where you store your medication and whether you store them in the pharmacy pill bottle or in another type of container.
         1. Prompt: Do you store your pills on your nightstand, in the bathroom, kitchen…? Do you keep your pills in the original bottle or use a 7-day pill case?
         2. Rationale: We want to find out in more depth about medication storage practices.
      2. How did you select where to store your medication and how did you develop a routine or habit to help you remember to take it?
         1. Prompt: how did you develop this strategy or storage location
         2. Rationale: we want to understand the factors that lead to someone’s adherence routine or storage routine as this is important to consider when designing an adherence device.
      3. In the past two weeks have you forgotten to take a medication? If yes, can you describe the circumstances and what you did? If no, have you ever forgotten to take your medication?
         1. Prompt: Has it been easy or hard for you to adhere to your medications with your current strategies? Are there factors like travel that impact your adherence?
         2. Rationale: With adherence generally reported at 50%, we want to know about self-reported adherence.
      4. Was it easy for you to adopt your current strategies, or did it take multiple attempts to achieve consistently take your medications?
         1. Prompt: did you have a strategy that you used before that didn’t work and lead to you adopting your current strategy?
         2. Rationale: we will see the process and steps leading to developing good medication adherence.
   3. Aids to Medication Adherence
      1. Do you currently use any objects like a toothbrush or water glass or a routine to assist you with taking your medications? Have you tried any in the past?
         1. Prompt: what do you use to help you adhere to your medications?
         2. Rationale: we will gain insight into some of the current ways that patients are trying to adhere to their medications
      2. Do you currently use or have used in the past any other product other than prescription bottles to store or dispense your medications? If yes, do you have a routine in place to manage how you store your medications?
         1. Prompt: have you ever used any digital or non-digital aids to help you with your medication adherence?
         2. Rationale: we will gain insight into some ways that patients have tried or are currently trying to adhere to their medications
      3. Do you use any reminder or alarm to help you remember to take your medications?
         1. Prompt: do you use a calendar, your apple watch or Alexa to remind you of your medications? Is it a regular alarm or a visual reminder?
         2. Rationale: we will gauge whether or not patients have used any sort of alarm as a reminder to take their medications
   4. Other People
      1. Is there anyone else in your household that helps or assists you with taking your medications (i.e. partners, children, parents, or friends)?
         1. Prompt: does someone you live with remind you every morning or set out your medication?
         2. Rationale: family or friend support or reminders can benefit someone’s medication adherence
      2. Is there anyone outside of your household that assists you with taking your medications?
         1. Prompt: Caretaker? Does someone drive to your house to refill your medication?
         2. Rationale: family or friend support or reminders can benefit someone’s medication adherence
      3. Are you responsible for helping anyone with their medications? If yes, how and why did you find yourself being responsible for that other person’s medications?
         1. Prompt: do you remind anyone to take their pills? Or refill someone’s pills?
         2. Rationale: investigating the role of co-adherence
   5. Medication Management
      1. Do you check for unused or expired medications? If so, do you dispose of them?
         1. Prompt: do you look at expiry dates or are you conscious about whether or not you have unused, old medication from a previous illness that you no longer need?
         2. Rationale: more medication clutter and disorganization may lead to less adherence for actual medication
      2. Have you ever felt stigmatized, judged, or perceived as an “ill” or “sick” person due to the way you take your medications? If an outsider sees you taking your medication or your pill bottles, do you feel concerned about their opinion or if they see you differently?
         1. Prompt: do you think people look at you differently knowing that you have to take daily medication? Are you unwilling to take medication in front of others?
         2. Rationale: we can look into the behaviors of patients and their attitudes towards their own pills
4. Changes to Routine or Lifestyle (Persistence):
   1. Health
      1. Have there been any significant changes to your health recently?
         1. Prompt: have you had any new diagnoses that have affected your daily life?
         2. Rationale: significant health changes may have a big impact on one’s medication adherence
      2. Have there been any changes to your medication and/or supplement list recently?
         1. Prompt: have you started any new medications? Or got rid of any?
         2. Rationale: a change to medication or supplements list could affect medication adherence routine or add to an overwhelming list of things to remember.
   2. Routine
      1. Have you had any business or vacation travels in the past year? If so, how did it impact the way you take your medications?
         1. Prompt: if you traveled, were you able to still take your medication on time each day?
         2. Rationale: travel is likely to interfere with medication adherence if patients can’t store their medication in their usual spot or take their normal pill dispenser
      2. Have you moved to a new location in the past few years? If so, how did it impact the way you take your medications?
         1. Prompt: If you moved houses or locations, did you have to change pharmacies or be further away from your doctor that caused you to be less adherent to refilling prescriptions? Did you have to move houses and your medication adherence was affected by a change of routine or set-up in your house?
         2. Rationale: a location change may prompt a change in medication adherence by changing routines or storage locations.
      3. Has the COVID-19 pandemic affected the way you take your medications? (i.e. by switching from remote to in-person work, etc.)
         1. Prompt: Are you at home more because of COVID and did this impact how you go about taking your medication?
         2. Rationale: COVID likely changed people’s daily habits and this could have affected their medication adherence.
   3. Other
      1. Have there been any other changes in your life that have not been brought up so far that have impacted the way you take your medications?
         1. Prompt: Have you faced any significant challenges that have not been mentioned that have impacted your adherence?
         2. Rationale: significant life changes may impact medication adherence.
      2. If your doctor or pharmacist gave you a free device to help you remember to take your medication, would you be interested?
         1. Prompt: Are you open to a trying something new if recommended?
         2. Rationale: Receptivity to our approach
5. Adherence in Other Health Domains:
   1. Have you ever been adherent to, or expressed interest in being adherent to, any other longitudinal health-related endeavors (i.e. an exercise program, diet, physical therapy, etc.) Have you ever been adherent to, or expressed interest in being adherent to, any long-term program like a diet, physical therapy, or exercise program?
      1. Prompt: do you have an exercise routine, do physical therapy, or follow a diet that you are fully adherent to or want to be adherent to?
      2. Rationale: we want to find out if some people treat other health measures differently, if some are not good at following a routine in general (adhering to anything) or are good about adhering to most things.
6. Demographic Information:
   1. How old are you? You can use a range like in my 50s, in my 60s, in my 70s, etc.
   2. What is your gender identity?
      1. Prompt: Female, Male, Other
   3. Which races/ethnicity do you consider yourself?
      1. Prompt: You may select one or more of the following: White, Black or African American, Hispanic or Latino, Asian, Other
   4. What is the highest level of education you completed?
      1. Prompt: Less than a high school diploma, GED, High school diploma, trade school, some college, Associate's degree, Bachelor's degree, Master's degree, Professional degree, Doctoral degree
   5. Can you describe how many people you live with? If you live with others, does that influence where you decide to place/store your medications?
   6. Do you live on one floor or multiple floors in your home?
   7. Would you describe your home location as urban, suburban, or rural?
7. Final thoughts
   1. We are considering designing a device that would use sensors to remind you only if you forgot your medication. For example, if you take a pill in the morning and missed it, you would be notified when you made coffee or stepped on your scale. Does this sound like something you would find helpful?
   2. Is there anything else you would like to tell us/share relating to the way you take your medications that we did not ask, or any comments or feedback for us?

Closing Statement: Thank you very much for your time, we truly appreciate your willingness to participate in this interview.
